# Phylogeography and Ecological Niche Modeling of the Alashan Pit Viper (*Gloydius cognatus*; Reptilia, Viperidae) in Northwest China and Adjacent Areas

**DOI:** 10.3390/ani13233726

**Published:** 2023-12-01

**Authors:** Rui Xu, Tatjana N. Dujsebayeva, Dali Chen, Byambasuren Mijidsuren, Feng Xu, Xianguang Guo

**Affiliations:** 1 Chengdu Institute of Biology, Chinese Academy of Sciences, Chengdu 610041, China; xurui201@mails.ucas.ac.cn; 2 University of Chinese Academy of Sciences, Beijing 100049, China; 3 Laboratory of Ornithology and Herpetology, Institute of Zoology CS MES RK, 93 al-Farabi Avenue, Almaty 050060, Kazakhstan; tatjana.dujsebayeva@zool.kz; 4 Department of Pathogenic Biology, West China School of Basic Medical Sciences and Forensic Medicine, Sichuan University, Chengdu 610041, China; chendali@scu.edu.cn; 5 Plant Protection Research Institute, Mongolian University of Life Sciences, Ulaanbaatar 210153, Mongolia; byambasuren.m@muls.edu.mn; 6 State Key Laboratory of Desert and Oasis Ecology, Xinjiang Institute of Ecology and Geography Chinese Academy of Sciences, Urumqi 830011, China; xufeng@ms.xjb.ac.cn

**Keywords:** phylogeography, ecological niche modeling, mitochondrial DNA, pit viper, Northwest China, last glacial maximum, Quaternary

## Abstract

**Simple Summary:**

Northwest China is characterized by unique geological and historical dynamics and endemic biota, but the joint influence of geography and past climate changes on the evolutionary history of endemic animals is poorly understood. We used two mtDNA genes (*ND4* and *Cytb*) and ecological niche modeling (ENM) to explore how the Quaternary climatic fluctuations and complex geography in Northwest China have shaped genetic diversity, genetic structure, and demographic history of the Alashan pit viper (*Gloydius cognatus*). Our results clearly show that the lineage diversification of *G. cognatus* is related to the expansions of deserts and/or the early Pleistocene integration of the Yellow River. The strong spatial genetic structure fits an isolation-by-distance model. The signature of demographic and range contractions during the last glacial maximum (LGM) is rejected by both mitochondrial evidence and ENM. In addition, the suitable habitat of *G. cognatus* is predicted to be decreased in the future, suggesting that conservation and management of evolutionary significant units (ESUs) should be a priority. Our findings provide the first insights on the evolutionary history of *G. cognatus* in arid Northwest China and adjacent areas throughout the Quaternary.

**Abstract:**

The joint impacts of historical geological events and Quaternary climatic oscillations in Northwest China on species evolution have been examined extensively in plant under a phylogeographic perspective. However, animal phylogeographic analyses in this region are still limited. The Alashan pit viper, *Gloydius cognatus*, occurs primarily in arid Northwest China and adjacent areas. Based on variation at two mtDNA genes (*ND4* and *Cytb*) in 27 individuals representing 24 populations, the spatial genetic structure and demographic history of *G. cognatus* were examined across its geographic range. Phylogenetic analyses revealed two well-supported allopatric clades (each with two distinct subclades/lineages), distributed across the southern (Qaidam Basin, Lanzhou Basin, and Zoige Basin [S1]; Loess Plateau [S2]) and northern (Ily Basin [N1]; Junggar Basin and Mongolian Plateau [N2]) regions. AMOVA analysis demonstrated that over 76% of the observed genetic variation was related to these lineage splits, indicating substantial genetic differentiation among the four lineages. A strong pattern of isolation-by-distance across the sampling populations suggested that geographic distance principally shaped the genetic structure. The four lineages diverged by 0.9–2.2% for the concatenated data, which were estimated to have coalesced ~1.17 million years ago (Mya), suggesting that the expansions of the Badain Jaran, Tengger, and Mu Us deserts during the Xixiabangma glaciation likely interrupted gene flow and triggered the observed divergence in the southern and northern regions. Subsequently, the early Pleistocene integration of the Yellow River and associated deserts expansion promoted the differentiation of S1 and S2 lineages (~0.9 Mya). Both mitochondrial evidence and ecological niche modeling (ENM) reject the signature of demographic and range contractions during the LGM for *G*. *cognatus*. In addition, ENM predicts that the suitable habitat of *G. cognatus* will contract in the future. As such, the conservation and management of ESUs should be a priority. Our findings provide the first insights on the lineage diversification and population dynamics of the Alashan pit viper in relation to geological history and Pleistocene climatic oscillations in arid Northwest China.

## 1. Introduction

Phylogeography may be the most useful tool for interpreting species responses to Quaternary climate changes from a genetic perspective. However, our current knowledge of Asian biogeography lags behind that of other regions, such as Europe and the Americas [1,2,3,4]. This is particularly true for snakes that have been useful for examining environmental and geological effects on phylogeographic structure because of their lower dispersal abilities and sensitivity to climatic fluctuations [5,6,7]. Indeed, limited information is currently available for terrestrial poikilotherms with narrow habitat requirements and limited dispersal potentials, such as venomous snakes whose secretive behaviors and toxicity to humans impede sample collection [8,9], and whose population density is declining [10].

Meanwhile, in recent years, phylogeographic studies in arid Northwest China have increased and mainly focus on the impact of the Quaternary climate fluctuations on species’ phylogeographic patterns, e.g., [11,12,13,14]. Notably, phylogeographic studies of plants in this region have sprung up like mushrooms after rain [11]. An increasing number of studies have shown that the deserts have an impact on the genetic structure and phylogeographic pattern of plant species, causing the speciation and population differentiation of many desert species [11,15,16].

Evidence from pollen records indicates that glaciation effects were not significant in the desert regions of Northwest China during the Quaternary [17]. However, glacial and interglacial cycles affected the evolutionary processes of species in this region, e.g., [15,18,19], through allopatric divergence, e.g., [10,20,21,22,23], range fragmentation, and regional range expansion [24,25]. Additionally, the uplift of the Qinghai–Tibetan Plateau (QTP) and global Pleistocene cooling promoted the formation and subsequent evolution of the deserts in North China [26,27]. As such, the desert zone may have acted as a geographic barrier that hindered gene flow among populations, which led to high genetic diversity among the populations and low genetic diversity within populations in arid Northwest China, e.g., [16,28,29,30,31]. However, the influence of historical climate oscillations on the evolutionary history of endemic animals in this region is poorly understood [32].

The *Gloydius halys–G. intermedius* species complex is a group of pit vipers widely distributed in the Palaearctic with 23 rows mid-back scales, which consists of 9 taxa of the Crotalinae subfamily (Viperidae family), including *G. halys*, *G. cognatus*, *G. caucasicus*, *G. caraganus*, *G. stejnegeri*, *G. rickmersi*, *G. shedaoensis*, *G. changaoensis*, and *G. intermedius* [33,34]. These pit vipers’ distribution area starts from Azerbaijan and Iran in the west and passes through several countries of Central Asia to Mongolia and China [35,36,37]. Although studies of this complex have involved many aspects, such as morphology, phylogeny, ecology, and captive breeding, our understanding is still limited about their diversity and evolution [37,38,39,40,41,42,43,44,45]. For instance, the Caucasian pit viper (*G. caucasicus*) has been elevated recently to species rank based on phylogeographic analyses and its four documented evolutionary significant units (ESUs) [33]; the Alay pit viper (*G. rickmersi*) was recently discovered and described from previously unnamed populations within this group in the strangest place from Kyrgyzstan [37], indicating more diversity than we previously recognized in this complex.

In the meantime, it should be noted that the Alashan pit viper, *G. cognatus*, was previously regarded as a synonym of *Gloydius brevicaudus* [46] or a subspecies of *G. halys* [37,47,48]. The Alashan pit viper, *G. cognatus*, was revalidated as a separate species by Shi et al. [42], and its validity was supported by Shi et al. [43]. Historically, the members of the *G. halys–G. intermedius* species complex in northern China were identified as *G. intermedius* [49,50]. Through morphological and molecular phylogenetic analysis, Shi et al. [42] found that the so-called “*G. intermedius*” represented three subspecies of *G. halys*, and elevated two of them to full species, namely *G. cognatus* and *G. stejnegeri*. According to Shi et al. [42], *G. cognatus* is mainly distributed in the vast desert or desertification grassland in Mongolia and Northwest China, and lives at an altitude of more than 1300 m or even more than 3000 m, thus, serving as an ideal species to evaluate the phylogeographic patterns of poikilotherm species in Northwest China and adjacent regions. Nevertheless, the genetic structure and evolutionary history of *G. cognatus* have not been rigorously investigated due in part to very limited sample size in previous studies [42,43]. The present study aims, therefore, to assess for the first time the genetic variation of the Alashan pit viper across its entire geographic range.

To complement genetic analysis, ecological niche modelling (ENM) constitutes a powerful set of tools for investigating organisms’ evolutionary patterns and processes [51], since they are linked to the variation in the abiotic environment (e.g., temperature, precipitation, and topography) [52]. These methods allow the inference of the environmental suitability of species [53], the reconstruction of paleo-distributions, and the testing of ecological niches similarity between species, and among other applications [54]. The combination of ENM with genetic studies may provide a better understanding of the evolutionary biology and geographic patterns of species and speciation.

In this study, through the combination of phylogeographic and ecological niche modeling analyses, we attempted to infer the spatial genetic structure and trace the population history of *G. cognatus* across its distribution range. Specifically, we aimed to (i) document the phylogeographic structure and the timing of genetic diversification within the Alashan pit viper; (ii) to evaluate the correlation between genetic distance, geographic distance, and environmental distance; (iii) to explore the relationship between historical demographic changes and past climate fluctuations.

## 2. Materials and Methods

### 2.1. Sample Collection

**Figure 1:**
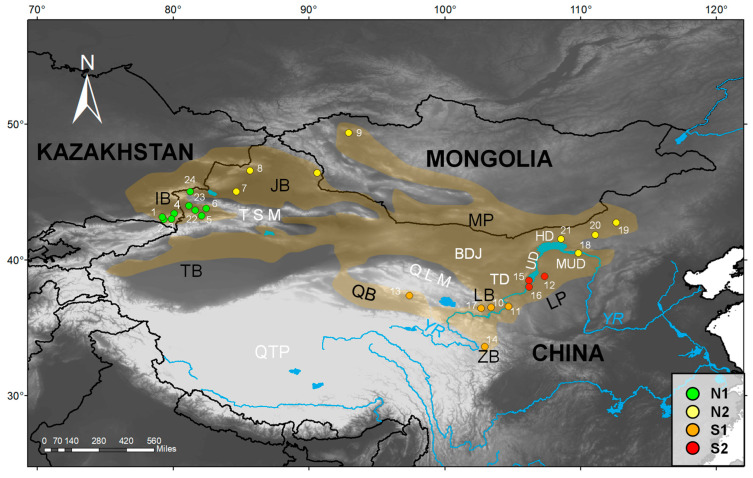
Collection sites for the samples of *G. cognatus* used in this study. Sites are numbered as in Table 1 and Table S1; subclades/lineages/phylogroups are highlighted by different colors referring to Figure 2. Sites 14–24 are retrieved from previous studies [42,43,55,56], of which sites 17, 20–24 are approximate due to a lack of precise coordinates. The background outlines the known distribution range of *G. cognatus* according to Shi et al. [42] and our field survey. The yellow circle without number indicates our recent field record of *G. cognatus* in Qinghe County, Xinjiang, China. BJD, Badain-Jaran Desert; LP, Loess Plateau; HD, Hobq Desert; IB, Ily Basin; JB, Junggar Basin; LB, Lanzhou Basin; MP, Mongolian Plateau; MUD, Mu Us Desert; QB, Qaidam Basin; QLM, Qilian Mountains; QTP, Qinghai-Tibetan Plateau; TB, Tarim Basin; TD, Tengger Desert; TSM, Tianshan Mountains; UD, Ulanbh Desert; YR, Yellow River; ZB, Zoige Basin.

This study relies on information retrieved from 27 individuals of *G. cognatus* coming from 24 localities. Among them, there were 13 new collected individuals from localities in northwestern China, southeastern Kazakhstan, and Mongolia; information from the other 14 individuals from 12 localities was also retrieved from previous studies [42,43,55,56]. In addition, one individual of *G. halys* collected from Mongolia was also included in this study as an outgroup. Based on the topography and landscape, we categorized the sampled sites a priori into seven geographic units: Ily Basin (IB), Junggar Basin (JB), Mongolian Plateau (MP), Qaidam Basin (QB), Lanzhou Basin (LB), Loess Plateau (LP), and Zoige Basin (ZB). Herein, MP is divided politically and geographically by the Gobi (desert) into the independent state of Mongolia (also called Outer Mongolia) in the north and the Inner Mongolia Autonomous Region of China in the south. Pit vipers were identified via morphological determination following the classification system of Shi et al. [42]. The captured snakes were euthanized with an overdose of sodium pentobarbital via intraperitoneal injection, and liver tissues were extracted and preserved in 95% ethanol following the animal-use protocols approved by Chengdu Institute of Biology (CIB), Chinese Academy of Sciences (CAS). Liver or scale tissues from samples were fixed with 95% ethanol and stored at −20 °C before DNA extraction. The voucher specimens were deposited in CIB, Institute of Zoology in Kazakhstan, and Xinjiang Institute of Ecology and Geography, CAS. To incorporate the publicly available data on *G. cognatus* and especially the data from Shi et al. [42], we amplified the mitochondrial NADH dehydrogenase subunit 4 (*ND4*) and cytochrome b (*Cytb*) genes in this study. A detailed list of information about all specimens is provided in Table 1 and the Supplementary Materials (Table S1). During the preparation of the manuscript, we collected two other specimens of *G. cognatus*, with voucher numbers of GXG2728 and GXG3335, from the Cha’gan’guole village (E 90.61, N 46.41, 1198 m a.s.l.) in Qinghe County, Xinjiang, China (see Figure 1, site without number in the Junggar Basin). We did not sequence the two specimens collected in Qinghe, although their occurrence record was used in ENM.

### 2.2. DNA Extraction, PCR Amplification and Sequencing

Genomic DNA was extracted with TIANamp Genomic DNA Kit (TIANGEN, Beijing, China) for 13 novel samples of *G. cognatus* and 1 sample of *G. halys* (Table 1). We amplified and sequenced two mitochondrial DNA (mtDNA) gene fragments—*ND4* and *Cytb*. A total of 831 base-pair (bp) fragments of the *ND4* gene were targeted for sequencing with the primers *ND4*/Leu [57]. A total of 1113 bp fragments of the *Cytb* gene were targeted for sequencing using the primers LAM2N/HAM [58]. The PCR reaction mixture contained 12.5 μL of 2 × EasyTaq SuperMix (Tsingke Biol-Tech, Chengdu, China), 0.2 μM of each primer, and 1–2 μL of genomic DNA (~50 ng) for a total volume of 25 μL. The thermal cycling was performed with initial denaturation for 4 min at 94 °C, followed by 35 cycles of 30 s at 94 °C, 30 s of annealing at 56.1 °C for *ND4* and 50.8 °C for *Cytb*, 54 s of elongation at 72 °C, and a final extension of 10 min at 72 °C. Amplified products were visualized on 1% agarose gels and were sequenced for double strands after being commercially purified by Sangon Biotech (Beijing, China) using the same primers as used for the PCR. Chromatograms, including sense and antisense, were edited and assembled using the SeqManII in DNASTAR 5.0 (DNASTAR, Madison, WI, USA) to obtain a single consensus sequence. All novel sequences were deposited in GenBank under accession numbers OR513115–OR513142 (Table 1).

### 2.3. Phylogenetic Analyses

A total of 135 sequences (70 for *Cytb*, 65 for *ND4*) were considered in this study, including 28 generated in this study and 107 available from previous studies, accessible in GenBank (Table 1 and Supplementary Materials, Table S1). The sequences were first aligned with MUSCLE in MEGA v7 [59] using the multiple alignment default parameters and corrected by hand. Then, the *ND4* and *Cytb* sequences were concatenated with the software SeaView v4 [60]. Sequences were further checked by eye, especially at the polymorphic sites, and translated into amino acids to assess their accuracy. Meanwhile, additional sequences of *ND4* and *Cytb* of *G. halys–G. intermedius* complex and outgroup species were downloaded from GenBank (Supplementary Materials, Table S1). Among them, sequences of 15 individuals of *G. cognatus* from GenBank were included in the dataset for phylogenetic analyses. By combining our newly determined sequences with the mtDNA data retrieved from GenBank, we generated a concatenated alignment dataset comprising 680 bp of *ND4* and 957 bp of *Cytb*. A total of 72 concatenated sequences were compiled from two outgroups and 70 individuals of the *G. halys–G. intermedius* complex. The alignment dataset for *G. cognatus* comprised 418 variable sites (169 in *ND4* and 249 in *Cytb*), of which 301 are parsimony informative, with 126 in *ND4* and 175 in *Cytb*.

Phylogenetic relationships within the *G. halys–G. intermedius* complex were inferred using the combined sequences of the *ND4* and *Cytb* genes. We used Bayesian inference (BI) and maximum likelihood (ML) with partitioned models to infer phylogenetic trees and assess nodal support. Based on the study of Shi et al. [42], *G. monticola* and *G. liupanensis* were selected as the outgroup taxa (Supplementary Materials, Table S1). PartitionFinder v2.1.1 [61] was used to determine the best nucleotide model and partitioning scheme for the dataset based on corrected Akaike information criterion (AICc). A summary of DNA partitions and relevant models as determined by PartitionFinder is given in the Supplementary Materials (Table S2). Bayesian inference analysis was carried out using MrBayes v3.2.6 [62]. Two simultaneous runs of 20 million generations were conducted for the dataset and trees were sampled every 1000 generations, with the first 25% discarded as burn-in. The runs were checked for convergence using the values of the potential reduction factor (PSRF; values close to 1.00) and effective sample size (ESS; values above 200) in Tracer v1.7.1 [63]. The first 25% of the trees were discarded as burn-in. A 50% majority-rule consensus tree and posterior probability values were computed, and nodes were considered robust when posterior probability (PP) PP ≥ 0.95. We generated the ML tree using IQ-TREE v1.6.7 [64] according to the best model for each partition determined by AICc. The reliability of the ML trees was estimated using the ultrafast bootstrap approach (UFBoot) with 5000 replicates, and nodes were considered to be well-supported with UFBoot ≥ 95% [65]. The phylogenetic trees were visualized using FigTree v1.4.4 [66].

Because intraspecific gene evolution cannot always be represented by a bifurcating tree, haplotype networks may more effectively portray the relationships among haplotypes within species. Therefore, we also constructed an unrooted parsimony haplotypes network for the *G. cognatus* with 25 concatenated sequences of *ND4* and *Cytb*. The samples ‘CHS098’ and ‘CHS825’ from Li et al. [56] were excluded due to lack of *ND4* sequences, and they were also not used in subsequent population genetic analyses. The TCS network approach as implemented in PopART v1.7 [67] was used to build the haplotype networks. This approach allows to present intraspecific evolution, and, thus, better recognizes relationships between populations [68].

### 2.4. Population Genetic Analyses

We used DNAsp v5.10 [69] to calculate nucleotide diversity (π) and haplotype diversity (*Hd*) for each clade (with the number of samples > 3) with the concatenated mtDNA dataset. To evaluate if significant genetic differentiation and genetic structure exist among lineages of *G. cognatus*, we used Arlequin v3.5.2.2 [70] to calculate the genetic differentiation coefficient (*F*_st_) among lineages, and we used MEGA to calculate the average genetic distance between lineages and within lineage with uncorrected p-distance. To estimate the source and significance of genetic variation, AMOVA analysis was conducted in Arlequin with three grouping strategies: (i) two groups based on North and South clades; (ii) three groups based on the northern clade, S1, and S2; (iii) four groups according to each lineage.

### 2.5. Testing Correlations between Genetic, Environmental, and Geographic Distances

To assess the relative contributions of geography and environment to the population differentiation, isolation-by-distance (IBD) [71] and isolation-by-environment (IBE) [72] tests were carried out for *G. cognatus*. Pairwise genetic distances (p-distances) between sampling sites with precise location coordinates (i.e., sites number 1–16, 18, and 19 in Figure 1) were estimated in MEGA (Supplementary Materials, Table S3). Euclidian geographic distances for the pairs of sampling sites were estimated in GeographicDistanceMatrixGenerator v1.2.3 [73]. Nineteen contemporary biological climate variables were downloaded from WorldClim v1.4 (http://www.worldclim.org (accessed on 15 August 2022)) [74]. The bioclimatic variables were extracted based on latitude and longitude data for the 18 sampling sites in ArcGIS v10.8. The highly correlated variables were removed (Pearson’s r > 0.80), and nine non-highly correlated environmental variables (Bio1, 2, 3, 7, 8, 12, 14, 15, and 19) were obtained for subsequent analysis. The environmental Euclidean distance between sample points was calculated by using the “vegan” package in R [75]. The relationships between genetic distance (p-distance) and logarithm of geographic distance (log km) as well as between genetic distance (p-distance) and environmental distance were calculated for the 18 sampling sites using Mantel tests in ZT v1.1 [76]. Meanwhile, to rule out potential spurious correlations from IBE and IBD effects, partial Mantel tests were conducted by controlling for constant geographic distances and environmental distances, respectively. In addition, geographic or environmental barrier between pairwise sampling points might affect the gene flow between populations, so the Tianshan Mountains and Qilian Mountains were chosen as indicators for partial Mantel tests. The test results were plotted with Microsoft Excel 2021.

### 2.6. Divergence Dating

To infer historical evolutionary processes, we used a Bayesian inference to estimate the time of divergence of major lineages using BEAST v1.8.4 [77] on our concatenated dataset. Due to a lack of reliable fossil evidence, we adopted a two-step method similar to that employed by Liu et al. [78]. First, to introduce the calibration points, 39 sequences of *ND4* and *Cytb* were retrieved from GenBank (Supplementary Materials, Table S1), which included two species of *Montivipera*, two species of *Macrovipera*, two species of *Vipera*, two species of *Sistrurus*, four species of *Crotalus*, three species of *Porthidium*, and six outgroups (*G. brevicaudus*, *G. blomhoffi*, *G. monticola*, *G. rubromaculatus*, *G. liupanensis*, and *G. tsushimaensis*). We then compiled the dataset to date the most recent common ancestor (MRCA) of *G. cognatus*. PartitionFinder was used to select the best partition and evolutionary models in our analysis based on AICc. Following Asadi et al. [33], three calibration points were chosen to estimate divergence times of *Gloydius*: (i) the divergence of three populations of the genus *Porthidium* in South America, some 3.5 million years ago (Mya), using a normal distribution model (mean = 3.5, SD = 0.51), (ii) the divergence between *Crotalus* and *Sistrurus* before 9 Mya, using a lognormal prior model with a zero offset of 9 Mya (mean = 1.0, SD = 1.0), and, finally, (iii) the divergence of the Eurasian vipers clade (genera *Macrovipera*, *Montivipera*, and *Vipera*) about 20 Mya, as suggested by fossil data, using a lognormal prior model with a zero offset of 17 Mya (mean = 1.0, SD = 1.0). Clock models and trees were linked, and a relaxed lognormal clock and birth–death process were implemented. In the second step, all sequences of *G. cognatus* were used to estimate the coalescent time of internal lineages according to the above age estimate for its MRCA (1.11 Mya, 95% highest probability density interval (HPD): 0.68–1.67 Mya), set as a normal distribution model (mean = 1.11, SD = 0.50). A relaxed lognormal clock and constant-size coalescent tree prior were adopted. We performed 10 million generations of MCMC chain and sampled every 1000 generations. Models, prior settings, and parameters are listed in the Supplementary Materials (Table S2).

### 2.7. Inference of Demographic Histories

We used three methods to infer variation in population size over time for the major clades of *G. cognatus*. First, we calculated the Tajima’s *D* [79], Fu’s *Fs* [80], and *R_2_* [81] implemented in DnaSP and their respective *p* values with 10,000 simulated samples. These statistics have high power to detect population expansion for mtDNA under a variety of different circumstances, either when the sample size is large (*Fs*) or when the sample size is small (*D*), and the number of segregating sites is low (*R_2_*). Second, we used the ‘mismatch distribution’ function in Arlequin to draw mismatch distribution (MD) plot. We also estimated the sum of square deviations (*SSD*) and the Harpending’s raggedness index (*Rg*) as well as their respective *p* values with 10,000 bootstrap replicates to evaluate hypotheses concerning recent population growth for each clade. Third, to estimate the shape of population growth over time, we constructed Bayesian skyline plots (BSP) [82] with BEAST. The nucleotide substitution model was obtained from ‘PartitionFinder2’ in PhyloSuite v1.1.15 [83]. We used the strict clock model and 0.0124/site/million years (95%HPD: 0.005–0.02) for the substitution rate which was obtained from the divergence dating described above. The substitution rate was set as a normal distribution model (mean = 0.0124, SD = 0.0025). Models, prior settings, and parameters are listed in the Supplementary Materials (Table S2).

### 2.8. Ecological Niche Modeling

To explore the impact of climate change on the potential distribution of *G. cognatus* since the Pleistocene and until 2070, we conducted ENM using MaxEnt v3.4.1 [84]. Maxent is one of the most commonly used methods for inferring species distributions and is particularly useful for presence-only data. Moreover, MaxEnt has turned out to be a reliable method when the sample size is relatively small [85]. Occurrence localities were compiled from the fieldwork and specimens used in this study (see above, Table 1). Additionally, we supplemented these data with localities that had associated genetic data from [42,43]. To remove spatial biases, we spatially filtered the dataset to ensure no two localities were within 10 km of one another [86] using SDMtoolbox v2.5 [87] in ArcGIS. A total of 19 georeferenced occurrences were used to run the models, which were from our field surveys and the published literature with precise coordinates (Supplementary Materials, Table S4). To approximate modeling assumptions regarding dispersal and biotic interactions more closely, we delimited a custom study region by drawing a minimum rectangle around the localities and adding a 10° buffer [88,89]. The area covers from 65–120° E and 25–60° N, which is larger than the current distribution of *G. cognatus*. We downloaded 19 climate variable factors for 5 different periods from the WorldClim database (http://www.worldclim.org) [75]: the contemporary period (present: 1960–1990), the last glacial maximum (LGM, ~0.02 Mya), the last interglacial period (LIG, 0.12–0.14 Mya), the mid-Holocene (MH, ~6000 years ago), and the future, namely the 2070s (averaged for 2061–2080). The spatial resolution of climate variable factors in the present and the LIG were all 30 arc-seconds, and others were all 2.5 arc-minutes. For the LGM and MH we chose the model for interdisciplinary research on climate (MIROC). MIROC6, under the Shared Socio-economic Pathways 245 and 585 (SSP 245/585), was employed for the 2070s. We addressed the level of correlation between the 19 bioclimatic variables for current conditions using SDM toolbox [87] in ArcGIS and kept those with low correlation (r < 0.8) to perform ENM. Nine environmental variables (Bio1, 2, 3, 7, 8, 12, 14, 15, and 19) were reserved (Supplementary Materials, Table S5) for subsequent analyses. We built all niche models based on climate data from the contemporary period, and then projected it to the other four periods. In Maxent, 70% of the distribution data were randomly selected as the training set and 30% as the test set for 100 bootstrap replicates. We used the ENMeval package [90] in R to manage model complexity and determine the optimal combination of MaxEnt feature classes and regularization multipliers. The optimal model had a regularization multiplier of 1.0 and a linear/quadratic features class. The remaining parameters were set as the default. To avoid over-extrapolation of the present and past ENM projections, we used a multivariate environmental similarity surface (MESS) analysis [91]. This analysis was conducted to check whether the estimated projections contained combinations of climatic variables not represented by the training dataset. The MESS analysis indicates the locations with analogous and non-analogous habitats in relation to the training points [91]. Negative MESS values indicate areas where the projection is more unreliable because of at least one variable being outside the range encountered during the calibration of the current model [91]. The area under the receiving operator characteristics curve (AUC) was used to evaluate the model reliability of the prediction results, ranging from 0.5 to 1.0, and AUC > 0.7 indicates a fair model. The potential suitable distribution area of each period was calculated in ArcGIS based on SDMtoolbox. The habitat suitability index ranged from 0–1; the larger the number, the higher the adaptability of the habitat, and the more suitable for the survival of G. cognatus. We divided the range of habitat suitability index as 0 ≤ colorless < 0.2, 0.2–0.4, 0.4–0.6, and 0.6–1.0, with colors from light to dark. The importance of each variable was assessed based on percent contribution values reported in MaxEnt’s output files.

## 3. Results

### 3.1. Phylogenetic Relationships

**Figure 2:**
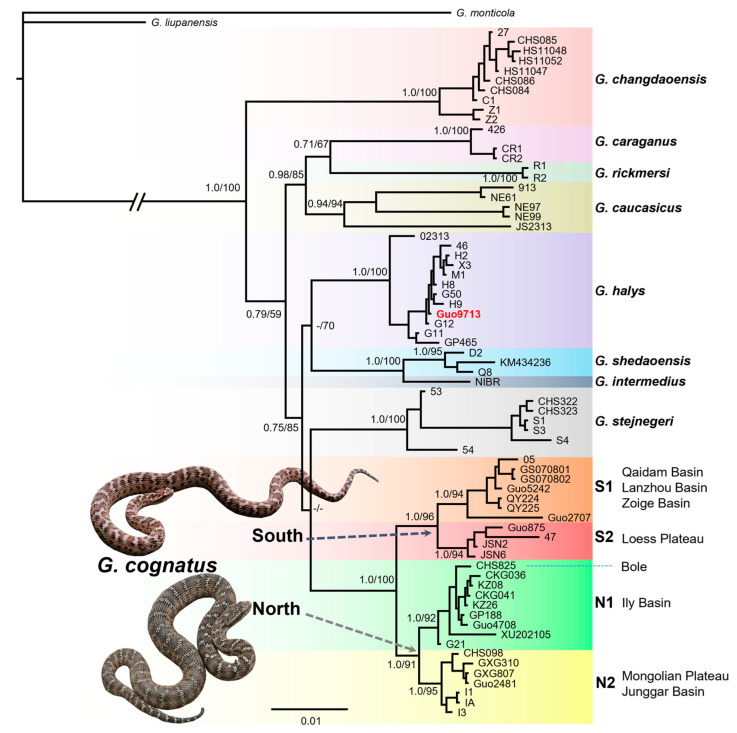
A majority-rule consensus tree inferred from Bayesian inference by using MrBayes v.3.2. The tip of the tree refers to the voucher number in Table 1 or code in Table S1. Different species are highlighted, and four lineages are identified in *G. cognatus*. Bayesian posterior probability (PP) and maximum likelihood UFBoot values are shown on major nodes. Dashes represent nodes with PP or UFBoot values lower than 0.5 or 50%, respectively. Snake photos by Xianguang Guo.

The tree topology was similar for the BI and ML analyses, and both indicated that pit vipers of the complex formed well-differentiated clades with high support values of PP and UFBoot, respectively (Figure 2). As expected, in our newly sequenced samples, only ‘Guo9713’ was clustered with *G. halys*, while the remaining 12 samples were allied to *G. cognatus*. All samples of *G. cognatus* formed a monophyletic group (PP = 1.0, UFBoot = 96) comprising two allopatric clades (North and South) with strong support. The samples in the well-supported Clade South (PP = 1.0, UFBoot = 100) consisted of two allopatric subclades (phylogroups/lineages): S1 across the Qaidam, Lanzhou, and Zoige basins; S2 across the Chinese Loess Plateau (Ningxia, Otag Banner in Inner Mongolia). The well-supported Clade North (PP = 1.0, UFBoot = 91) was also composed of two allopatric subclades/lineages: N1 covered the Ili Basin in Xinjiang and southeast Kazakhstan, while N2 contained the Junggar Basin and Mongolian Plateau (Inner Mongolia and West Mongolia). Overall, the four lineages corresponded to well-defined geographic regions, and no haplotype was shared among the different geographic regions.

**Figure 3:**
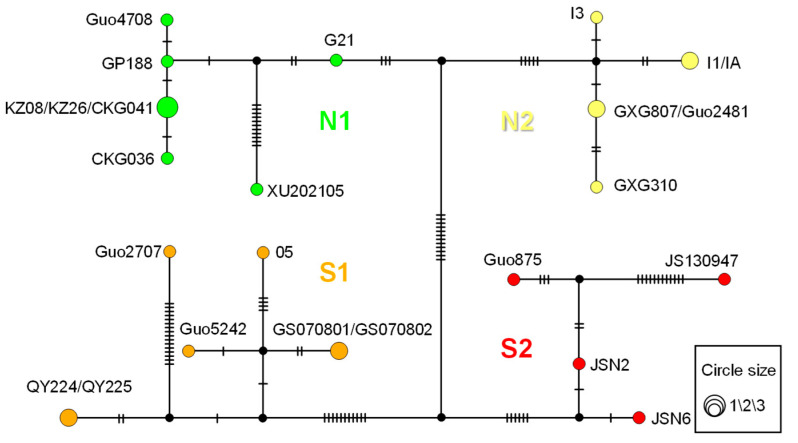
TCS network of the haplotypes inferred from a total of 25 concatenated sequences of *ND4* and *Cytb* for *G. cognatus*. In the network, colored circles indicate sampled haplotypes; black circles indicate vectors inferred by PopART software. Different filled colors represent the corresponding geographical origin from which the haplotype was sampled. Circle size corresponds to relative numbers of individuals sharing a particular haplotype. Short bars crossing network branches indicate substitution steps.

To obtain additional insight into the relationships among the individuals of *G. cognatus*, we analyzed our dataset, using the coalescent-based statistical parsimony network approach. As shown in Figure 3, the haplotype network recovered four main haplogroups, corresponding to the four subclades of the phylogenetic tree, respectively. Overall, the four haplogroups show high heterogeneity with geographic specificity. We identified 19 haplotypes, 6 of which came from the Ily Basin (N1), 4 from the Junggar Basin and Mongolian Plateau (N2), 5 from Qaidam, Lanzhou and Zoige basins (S1), and 4 from the Loess Plateau (S2). Having an advantage over the bifurcating tree in detail at the intraspecific level, the haplotype network could intuitively reflect the genetically greater distances between ‘XU202105’ and other haplotypes in the phylogroup N1 (>12 substitution steps). Similarly, in the phylogroup S1, ‘Guo2707’ was distantly related to the remaining haplotypes, with more than 18 substitution steps. In the phylogroup S2, ‘JS130947’ was distantly related to the other three haplotypes.

### 3.2. Genetic Diversity and Genetic Structure

Overall, *G. cognatus* harbors high haplotype diversity (0.9767) and low nucleotide diversity (0.01427) (Table 2). Nucleotide diversity ranged from 0.00172 (N2) to 0.00638 (S2) among the four lineages. Haplotype diversity ranged from 0.8667 (N2) to 1.0000 (S2) throughout the four lineages.

The fixation index analysis indicated that the *F*_st_ values among the four lineages ranged from 0.616 (S1 and S2) to 0.809 (S1 and N2) (Table 3). The significance test among the four lineages was significant (*p* < 0.01), indicating significantly high genetic differentiation, as well as a low genetic divergence among the four lineages (Table 3), with a p-distance ranging from 0.9% (N1 and N2) to 2.2% (S1 and N2). The largest genetic distance within each lineage was that of S1 and S2 (0.6%), and the smallest one within each lineage was that of N1 (0.2%). The results implied highly genetic homogeneity among populations within each lineage.

The results of AMOVA are shown in the Supplementary Materials (Table S6). In all three grouping strategies, significant genetic variation was mainly among groups (59.25%, 69.49%, and 76.71%, respectively). When all individuals were divided into four groups according to lineage, the fixation index *Fct* between groups accounted for the largest variation (76.71%, *p* < 0.001).

### 3.3. Geography and Environment Impact on Genetic Structure

The results of the IBD and IBE analysis are shown in the Supplementary Materials (Figure S2). For the IBD analysis, after the logarithmic transformation of geographic distance, the Mantel test indicated that the genetic distance was significantly related to geographic distance (*R²* = 0.2438, *p* = 0.001), in accordance with the IBD pattern. The partial Mantel test showed that when the geographical barrier (Tianshan and Qilian mountains) was controlled, the genetic distance was significantly correlated with the geographic distance (*r* = 0.413, *p* < 0.01). When geographic distance was controlled, genetic distance had no significant correlation with the geographic barrier (*r* = 0.1059, *p* > 0.05), which revealed that geographic barriers may have little effect on the genetic differentiation of *G. cognatus*.

The result of the Mantel test for the IBE (Supplementary Materials, Figure S1) indicated that genetic distance had no significant correlation with environmental distance (*R²* = 0.1548, *p* > 0.05). A partial Mantel test showed that when environmental distance was controlled, genetic distance and geographic distance were significantly correlated (*r* = 0.4802, *p* < 0.05). When geographic distance was controlled, the correlation between genetic distance and environmental distance was not significant (*r* = −0.0827, *p* > 0.05). The results revealed that geographic distance played a greater role in the genetic differentiation of *G. cognatus* than environmental distance.

### 3.4. Divergence Time and Historical Demographic Change

**Figure 4:**
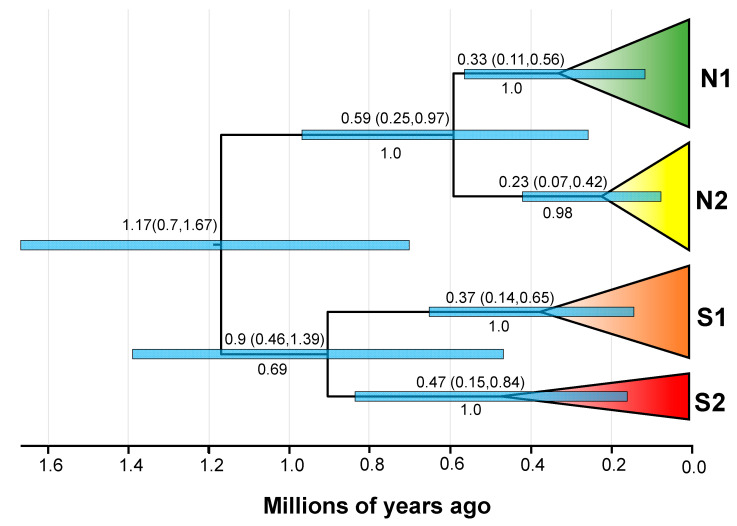
Molecular dating of *G. cognatus* based on the concatenated *ND4* and *Cytb* genes. Divergence dates with 95% HPD and posterior probability (PP) for major nodes are shown.

Our molecular dating analyses revealed that *G. cognatus* separated from other species of the *G. halys–G. intermedius* complex in the early Pleistocene (2.15 Mya, with 95% HPD of 1.42–2.99 Mya) (Supplementary Materials Figure S2). As shown in Figure 4, the MRCA of *G. cognatus* was dated in the early Pleistocene (~1.17 Ma; 95% HPD: 0.7–1.67 Mya). Accordingly, divergence between Clade South and Clade North commenced at ~1.17 Mya. After that, splits occurred within these two clades. The divergence between S1 and S2 lineages happened at 0.9 Mya (95% HPD: 0.49–1.39 Mya), and then the divergence between N1 and N2 lineages occurred at 0.59 Mya (95% HPD: 0.25–0.97 Mya) during the mid-Pleistocene.

**Figure 5:**
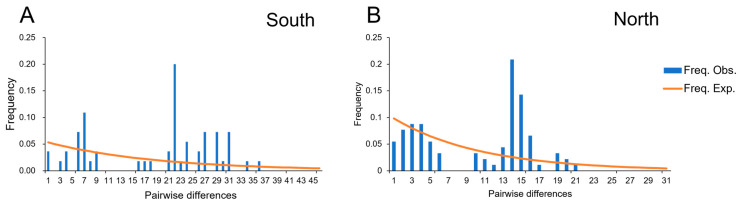
Results of the mismatch distribution analysis of each clade. (**A**) South, the southern clade; (**B**) North, the northern clade. Blue bars represent the pairwise differences of the observed distribution, and orange lines represent the theoretically expected distribution under a population expansion model.

**Figure 6:**
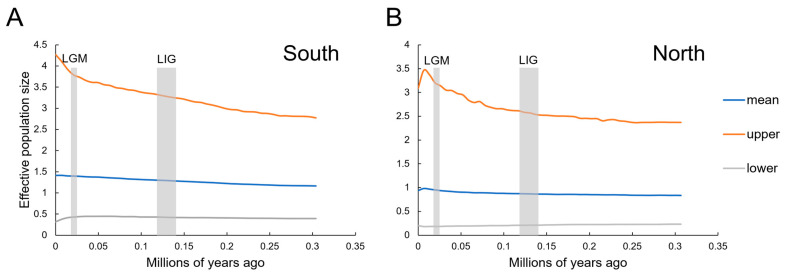
Bayesian skyline plots of each clade. (**A**) South, the southern clade; (**B**) North, the northern clade. The *y*-axes represent the estimated effective population size on a log-scale (*Ne* × τ/10^6^, the product of the female effective population size and generation length in years); *x*-axes represent time in millions of years ago (Mya). The vertical grey bar represents the duration of the last glacial maximum (LGM) and the last interglacial (LIG), respectively.

For neutrality tests, both clades exhibited similar results for *R_2_* albeit with different results for Tajima’s *D* and Fu’s *Fs* (Table 4). For Clade South, the values of Tajima’s *D* and Fu’s *Fs* were negative and statistically significant, suggesting recent demographic expansion. This is also supported by the significantly small positive *R_2_* value. As shown in Figure 5, both clades were characterized by multimodal, ragged, and erratic MDs, while both exhibited non-significant *SSD* and *Rg* values, implying a scenario of demographic growth (Table 4). As shown in Figure 6, the BSP of both clades suggested that the effective population size has remained stable in history, and the population expanded slightly since 0.25 Mya (Figure 6), and also kept growing in the LGM (~0.02 Mya). Specifically, the effective population size of Clade North had a slight decline during the Holocene.

### 3.5. Temporal Changes in Suitable Distributional Areas

One temperature variable, annual mean temperature (Bio1, 55.8%), and one precipitation variable, precipitation of coldest quarter (Bio19, 29.2%), were the variables that most contributed to the distribution of the species (Supplementary Materials, Table S4). This indicated that temperature and humidity played an important role in the potential geographic distribution pattern of *G. cognatus*. The response curves between the environmental variables and the prediction changes in the occurrence are shown in Figure S3. There was a non-linear relationship between the probability of occurrence and Bio1. The probability of occurrence peaked when bio1 was 5 °C. The response curve for occurrence also showed a clear non-linear relationship between the probability of occurrence and Bio19 (Supplementary Materials, Figure S3). The probability of occurrence decreased substantially when Bio19 ranged from 0 mm to 200 mm. The examination of the response curve profiles for these variables indicates that *G. cognatus* occurs in temperate areas with low levels of precipitation.

**Figure 7:**
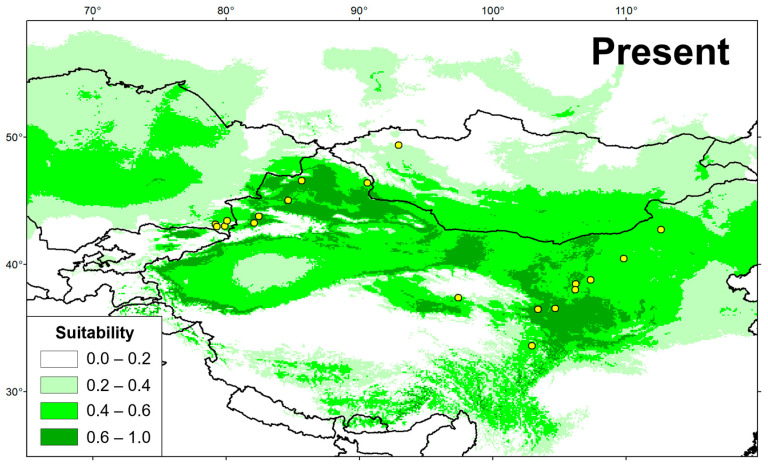
Potentially suitable distribution area obtained with MaxEnt for *G. cognatus* at present. Colorless area indicates low habitat suitability; green indicates a suitable habitat.

**Figure 8:**
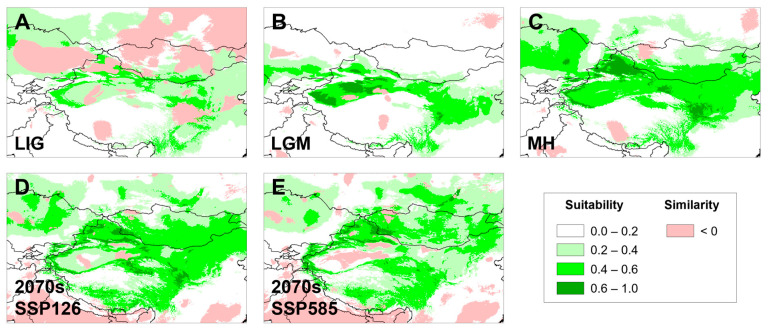
Potentially suitable distribution area in five different periods for *G. cognatus*. (**A**) LIG, last inter glacial; (**B**) LGM, last glacial maximum; (**C**) MH, mid-Holocene; (**D**) SSP 126 for 2070s, Shared Socio-economic Pathway 126; (**E**) SSP 585 for 2070s, Shared Socio-economic Pathway 585 for 2070s. Negative MESS scores, shown as similarity < 0 by pink, indicate areas without current equivalents of climatic conditions.

The AUC values for training and testing datasets were 0.909 ± 0.04 and 0.851 ± 0.06, respectively, representing the good credibility of simulation results. In the present day (Figure 7), the highly suitable habitat (0.6–1.0) of *G. cognatus* is mainly in the arid region in Northwest China, with a small amount in Kazakhstan and Mongolia, generally between the QTP and the Altay Mountains, which is overall consistent with the sample collection information. The total highly suitable habitat area (HSHA) is 8.58 × 10^5^ km^2^. During the LIG, the analysis did not support the existence of regions with a probability of distribution greater than 0.6 for *G. cognatus* (Figure 8). In the LGM period, the area of the HSHA was mainly concentrated in the Tarim Basin, with an area of 3.30 × 10^5^ km^2^ in total (Figure 8). The mid-Holocene was the warmest period, with the area of the HSHA increasing to 5.73 × 10^5^ km^2^ and mainly transferred to the Junggar Basin (Figure 8). In the 2070s, with climate change, whether in the SSP 126 or SSP 585 scenarios, the HSHA of *G. cognatus* will significantly decrease, mainly manifested by the disappearance of the HSHA in the Loess Plateau and the fragmentation of habitats in other regions. Under the SSP126 situation, the total HSHA is 4.75 × 10^5^ km^2^; it is even less under the SSP 585 situation, at 3.16 × 10^5^ km^2^ (Figure 8).

Projection uncertainty and areas with non-analog climates were assessed using MESS as a quantitative measure. Overall, the MESS analyses showed that climatic conditions were analogous between the current conditions and the different scenarios, except the LIG (Figure 8).

## 4. Discussion

### 4.1. Allopatric Divergence in the Ice Age

In the present study, our phylogenetic analysis from 27 individuals of *G. cognatus* recovered 2 well-supported clades of north and south, as well as 4 genetic lineages (Figure 2). Estimated divergence times of the genetic lineages of *G. cognatus* coincide with several early Pleistocene glacial cycles in China [92]. We dated the divergence time between the two clades back to ~1.17 Mya (95% HPD: 0.7–1.67 Mya), which falls within the early Pleistocene. Based on marine oxygen isotopic stage (MIS) 20–36 data, the earliest glaciation in China, the Xixiabangma glaciation (1.17–0.8 Mya), occurred during this period, and the temperature was much colder than that during the LGM [93,94]. During this period, northern China experienced a severe climate aridification and the temperature dropped sharply. The modern-like desert landscape in the Badain Jaran, Tengger Desert, and Mu Us deserts was likely established at around 1.2–0.9 Mya [21,27,95,96]. Given that *G. cognatus* is highly adapted to the stony gravel environment, the sandy deserts that expanded during the glacial age may have blocked the gene flow between the north and south populations. Overall, given the large divergence time ranges and the high morphological similarity among the clades/lineages, the separation within *G. cognatus* is fairly young, and secondary contact between these two clades cannot be excluded.

Meanwhile, the secondary diversification between the S1 and S2 lineages was dated to ~0.9 Mya (95% HPD: 0.49–1.39 Mya), falling within the time frame of the boundary of Xixiabangma glaciation and the maximum glaciation (Naynayxungla glaciation, 0.72–0.5 Mya), with the latter corresponding to MIS 14–18. During the maximum glaciation, there were two large cold periods in the Zoige Basin, which can be compared with the two glacial stages of Naynayxungla of Mt. Xixiabangma Region in the Himalayas [93]. Based on the distributional patterns of the S1 and S2 lineages, their divergence may be related to the environmental heterogeneity between the Loess Plateau and the Lanzhou and Zoige basins.

Subsequent differentiation of the N1 and N2 lineages was dated to ~0.59 Mya (95% HPD: 0.25–0.97 Mya), falling with the time frame of the Naynayxungla glaciation. Lineage N2 comprises the samples from the Junggar Basin and Mongolian Plateau. As we know, the Junggar Basin is located in northern Xinjiang, and it includes Chinese and Mongolian parts, while the latter is restricted to the Barun-Khure Basin. On the one hand, the Junggar Basin is bounded by Dzungarian Alatau, Tarbagatay, and Saur mountains at the west, Tianshan Mountains at the south, and Altay Mountains at the north. On the other hand, there is one passage connecting it with arid areas of northern China and southern Mongolia (eastern). Thus, pit vipers could migrate between the Junggar Basin and adjacent areas despite the partial isolation of this region by mountains.

The divergence of the N1 lineage may be associated with the long-standing (since the Late Pliocene) isolated position of the Ily Basin due to mountainous surroundings and a specific relatively temperate semi-arid climate, dominated by the penetration of westerly winds [97]. Moreover, recent studies have suggested that the Ily Basin may be a plausible refugium for desert herpetofauna in Central Asia [78,98,99]. Meanwhile, it should be noted that a sample (CHS825) from Bole (the Junggar Basin) is embedded in the population from the Ily Basin (number 24 in Figure 2). Nonetheless, this phenomenon does not seem to be exceptional. For instance, the population of the steppe racerunner (*Eremias arguta*) from Bole (Xinjiang, China) is phylogenetically nested within the Ily Basin group, belonging to an undescribed subspecies of *E. arguta* [100]. This finding implies that the Alatau, Kolguqin, and Borokonu mountains may not constitute a significant geographic barrier to the gene flow of the Alashan pit viper populations in the Ily Basin and Bole. This highlights the importance of using nuDNA, preferably in the form of multi-locus approaches (e.g., ddRAD), to infer introgression processes between mtDNA lineages and to further complete the inferences on the evolutionary history of these species.

### 4.2. Genetic Structure and Diversity

The null hypothesis of panmixia for the Alashan pit viper can be rejected based upon the heterogeneous geographic distribution of two mitochondrial clades and four lineages. The genetic heterogeneity within the distribution range is also supported by a high (*Fct* = 55.92% or 74.57%) and significant (*p* < 0.001) portion of the genetic variance among the two clades or four lineages (Supplementary Materials, Table S6). In addition, the genetic differentiation index (*F*_st_) between the main evolutionary lineages ranges from 0.616 to 0.809 (Table 3). According to Wright [101], an *F*_st_ greater than 0.25 indicates high genetic differentiation among the four lineages of *G. cognatus*.

In the present study, the distinct distribution of the two clades and four lineages across the distribution range (Figure 1) suggests that IBD played a determinant role in modeling the structure of *G. cognatus*, as evidenced by the positive correlation of genetic differentiation and geographic distance (Supplementary Materials, Figure S1). This pattern is likely due to limited gene flow among geographically distant populations. As shown by IBD and IBE analyses, the genetic differentiation within *G. cognatus* is mainly attributed to geographic distance, while environmental factors had little effect on genetic differentiation of *G. cognatus*. Meanwhile, the partial Mantel test suggests little influence of geographic barriers, such as the Tianshan and Qilian mountains, on the genetic differentiation of *G. cognatus*. The application of the Mantel test and partial Mantel test gives us the opportunity to offer a plausible mechanism to explain the heterogeneous distribution of clades in a south–north clime within arid Northwest China and adjacent areas.

Our haplotype network shows distinct geographic distribution patterns of genetic diversity in four major haplogroups, which correspond to the four major genetic units recovered in the phylogenetic tree (Figure 3). In Clade South, ‘Guo2707’ of S1 and ‘JS130947’ of S2 are separated from other haplotypes with more mutation steps in their respective lineages. With regard to geographic distribution, the S1 lineage is mainly distributed to the left bank of the Yellow River (YR), while ‘Guo2707’ occurs to the right bank (Site 11, Jingyuan County, Gansu). In contrast, only ‘JS130947’ in the S2 lineage is located to the left bank of the YR (Site 15, Yinchuan, Ningxia). Although there is still no agreement on the timing of the integration of the YR, multiple lines of evidence support early Pleistocene integration of the YR (e.g., ~1.7 Mya at Linxia [102] and Lanzhou [103], and 1.4–1.6 Mya in the Loess Plateau [104]). Given that the estimated coalescent times of S1 or S2 (0.37 Mya or 0.47 Mya) (Figure 4) is much younger than the ages for the integration of the YR, we suggest the intra-lineage variations may not be related directly to the integration of the YR. To test the barrier effect of the YR, further research should be conducted with more samples by integrating a landscape genetics approach.

### 4.3. Historical Population Demography

The Quaternary glacial period played an important role in the geographic structure and genetic diversity of species distribution [1,17,105]. Although arid Northwest China was not directly covered by glaciers [106], geological events and past climate changes have influenced the distribution pattern of plants and animals significantly [11,13,14,15,79,107].

Combined with the results of neutrality tests and mismatch distribution, the population size of both northern and southern clades of *G. cognatus* remained stable recently, without substantial expansion. The results of the BSP analysis also suggest the same trend that the effective population size has only slightly increased since 0.3 Mya, which corresponds to Guxiang (the penultimate) glacial period on the QTP during the Quaternary. Slight expansion instead of shrinkage occurred for *G. cognatus* in the penultimate glaciation (Figure 6), indicating that the penultimate glaciation did not limit the growth of local organisms.

In addition, the results of our ENM (Figure 8) fit the pattern of a rather stable population size as recovered with our genetic data. In all the past scenarios, there were rather small variations in the suitable space for the species occurrence. Even in the LGM, a period of extreme aridity in our study area, the suitable space was similar to current times, which rejects the signature of range contractions during the LGM for *G. cognatus*.

### 4.4. Conservation Implications

Within the *G. halys–G. intermedius* complex, *G. shedaoensis* is listed as vulnerable (VU) by the International Union for Conservation of Nature (IUCN), and little attention has been paid to the conservation of other species. The results presented in this study provide a framework for designing proper conservation and management guidelines for the Alashan pit viper populations. The analyses of mtDNA sequences of the Alashan pit viper suggest the existence of two geographically structured mitochondrial clades, with two subclades/lineages recognized in each clade. The pairwise genetic distance between the four lineages within *G. cognatus* (Table 3) was close to that (1.5–2.9%) among the four ESUs of *G. caucasicus* [33]. Thus, the four allopatric lineages in *G. cognatus* can be defined as distinct ESUs according to Moritz criterion of reciprocal monophyly of mtDNA alleles [108] as well as under the adaptive evolutionary conservation concept [109] as lineages demonstrating highly restricted gene flow from other such lineages within the higher organizational level (lineage) of the species.

Under the influence of climate change, based on ENM, we predict that the HSHA of *G. cognatus* will be reduced and fragmented to a considerable extent in the next 50 years, especially under SSP 585 with extreme carbon emissions. Moreover, the suitable habitat of *G. cognatus* will be drastically lost due to anthropogenic effects, including agricultural and husbandry development, overgrazing, and/or destruction of the Gobi and rocky deserts. Accordingly, under the joint effects of climate change and anthropogenic activities, the population size of *G. cognatus* is likely to decrease substantially in the near future. In such cases, the conservation and management of ESUs should be a priority [110].

It has been documented that various pit vipers of the *G. halys–G. intermedius* complex are specifically adapted to different habitats [42,50], and *G. cognatus* prefers rock and gravel habitats (our field observation). The plausible strategy to conserve the genetic diversity of *G. cognatus* is to protect the unique ecosystem of stony gravel habitats; the establishment of desert nature reserves may be one of the best solutions. In the meantime, *G. cognatus* is one of the well-known venomous snakes that causes livestock damage in northwestern China. It has been reported that the LD50 of *G. cognatus* (historically, so-called *Agkistrodon intermedius* [= *G. intermedius*]) from Xinyuan County in Xinjiang to mice is 0.38 mg/kg [111], and *G. cognatus* is recognized as the most toxic species of *Gloydius* in China. Thus, the conflict between *G. cognatus* and herdsmen should also be considered when implementing the protection strategy. Reasonable planning of the range of pastoral areas and management of grazing is necessary to reduce the casualties and damages caused by contact between herdsmen and *G. cognatus*.

## 5. Conclusions

This study explores for the first time the genetic differentiation within *G. cognatus*, with the recognition of two major clades in northern and southern regions, as well as four distinct lineages distributed in different geographic landscapes. Significant genetic differentiation dominated by geographic distance exists between the four lineages. The divergence between the southern and northern clades is likely triggered by the expansions of the Badain Jaran, Tengger, and Mu Us deserts during the Xixiabangma glaciation in China. Both mtDNA evidence and ENM reject the signature of demographic and range contractions during the LGM for *G. cognatus*. The ENM also supports that the suitable habitat of *G. cognatus* will contract and be concentrated in the Junggar Basin in the future. Thus, plausible conservation measures should be undertaken in a timely manner. We recall that our study is based on only mtDNA fragments and inadequate samples, and future studies should undoubtedly focus on population genomics with more extensive sampling and sophisticated analyses.

## Figures and Tables

**Table 1:** Sample information from the newly determined mtDNA data in this study. Abbreviations of geographic origin refer to Figure 1.

Species	Site Number	Voucher Number	Geographic Origin	Locality	Latitude	Longitude	Altitude (m)	GenBank Accession Number	
								*ND4*	*Cytb*
*G. cognatus*	1	KZ26	IB	Raiymbek District, Almaty Region, Kazakhstan	43.16	79.21	1774	OR513115	OR513141
*G. cognatus*	2	KZ08	IB	Raiymbek District, Almaty Region, Kazakhstan	42.97	79.31	1847	OR513116	OR513140
*G. cognatus*	3	CKG041	IB	Raiymbek District, Almaty Region, Kazakhstan	43.02	79.89	2065	OR513126	OR513130
*G. cognatus*	4	CKG036	IB	Uygur District, Almaty Region, Kazakhstan	43.43	80.07	1361	OR513127	OR513129
*G. cognatus*	5	XU202105	IB	Gongliu County, Xinjiang, China	43.26	82.09	1263	OR513128	OR513142
*G. cognatus*	6	Guo4708	IB	Nilka County, Xinjiang	43.80	82.43	1084	OR513121	OR513134
*G. cognatus*	7	Guo2481	JB	Wusu City, Xinjiang, China	45.05	84.66	965	OR513123	OR513132
*G. cognatus*	8	GXG310	JB	Hoboksar, Xinjiang, China	46.58	85.65	1120	OR513118	OR513138
*G. cognatus*	9	GXG807	MP	Tsalgar, Uvs, Mongolia	49.37	92.93	1140	OR513117	OR513139
*G. cognatus*	10	Guo5242	LB	Yongdeng County, Gansu, China	36.49	103.4	1880	OR513120	OR513135
*G. cognatus*	11	Guo2707	LB	Jingyuan County, Gansu, China	36.55	104.68	1538	OR513122	OR513133
*G. cognatus*	12	Guo875	LP	Otog Banner, Inner Mongolia, China	38.79	107.34	1337	OR513124	OR513136
*G. cognatus*	13	GS070802	QB	Delingha City, Qinghai, China	37.38	97.40	3000	OR513125	OR513131
*G. halys*	–	Guo9713	MP	Orgil, Khovsgol, Mongolia	48.60	99.32	1610	OR513119	OR513137

**Table 2:** Summary statistics of genetic diversity for each lineage in *G. cognatus*. *n*, number of samples; *h*, number of haplotypes; *S*, number of segregating sites.

Lineage	*n*	*H*	*S*	*Hd*	π
N1	8	6	17	0.8929 ± 0.111	0.00306 ± 0.00132
N2	6	4	6	0.8667 ± 0.129	0.00172 ± 0.00034
S1	7	5	30	0.9048 ± 0.103	0.00614 ± 0.00222
S2	4	4	20	1.0000 ± 0.177	0.00638 ± 0.00213
Total	25	19	84	0.9767 ± 0.018	0.01427 ± 0.00103

**Table 3:** The mean of uncorrected pairwise distances (p-distances) between lineages (below the diagonal) and within each lineage (in the diagonal with bold text) as well as the pairwise genetic differentiation *F*_st_ and its significance between lineages (above the diagonal) for *G. cognatus*.

Lineage	N1	N2	S1	S2
N1	0.002	0.721 **	0.798 ***	0.779 ***
N2	0.009	**0.003**	0.809 ***	0.797 ***
S1	0.021	0.022	**0.006**	0.616 ***
S2	0.017	0.019	0.016	**0.006**

Significance level: 0.01 **; 0.001 ***.

**Table 4:** Neutrality tests of the southern and northern clades for *G. cognatus*.

Clade	Tajima’s *D*	Fu’s *F_S_*	*R_2_*	*SSD*	*Rg*
	(*p*-Value)	(*p*-Value)	(*p*-Value)	(*p*-Value)	(*p*-Value)
South	−0.3224 (0.31)	0.4234 (0.544)	0.1609 ***	0.0515 (0.11)	0.1117 *
North	0.0266 (0.547)	−0.6367 (0.366)	0.1470 ***	0.0448 (0.11)	0.0477 (0.37)
All	−0.0128 (0.571)	−1.0839 (0.320)	0.1218 ***	0.0148 (0.35)	0.0168 (0.26)

Significance level: 0.05 *; 0.001 ***.

## Data Availability

The data supporting the results of this study can be found in the manuscript. All sequences generated during this study have been deposited in GenBank (https://www.ncbi.nlm.nih.gov/genbank/, accessed on 25 September 2023).

## Supplementary Materials

The following supporting information can be downloaded at: https://www.mdpi.com/article/10.3390/ani13233726/s1, Figure S1: Results of the Mantel test of IBD (A) and IBE (B) analysis for 18 sampling sites with precise location coordinates. Red lines show the trend of scatter plots. Figure S2: Molecular dating of the *G. halys–G. intermedius* complex based on the concatenated *ND4* and *Cytb* genes. Branch support values are not given for most intra-generic nodes to preserve clarity. C1, C2, and C3 indicate the calibration points placed on nodes, and red star denotes the node of *G. cognatus*. Figure S3: Average response curve profiles for the used variables in ENM. Table S1: Sample information of the mtDNA data retrieved from GenBank. Table S2: Data on the phylogenetic, molecular dating, and BSP analyses, including partitions, models, and parameters. Table S3: The 18 sampling sites used for IBD and IBE analyses. Table S4: Locations and coordinates used for ecological niche modeling. Table S5: Uncorrelated climatic factors used in ENM and their contribution rates. Table S6: Hierarchical analysis of AMOVA for *G. cognatus*. References [112,113,114,115,116,117,118,119,120,121,122,123,124,125,126] are cited in Table S1.
