# Supplementary material for: Phylogeography and Ecological Niche Modeling of the Alashan Pit Viper (Gloydius cognatus; Reptilia, Viperidae) in Northwest China and Adjacent Areas
Source: Animals (Basel). 2023 Dec 1;13(23):3726. doi: 10.3390/ani13233726 (PMC10705372; doi:10.3390/ani13233726)
Supplement: Supplementary file 1 [file animals-13-03726-s001.zip › animals-2748099-supplementary.pdf]

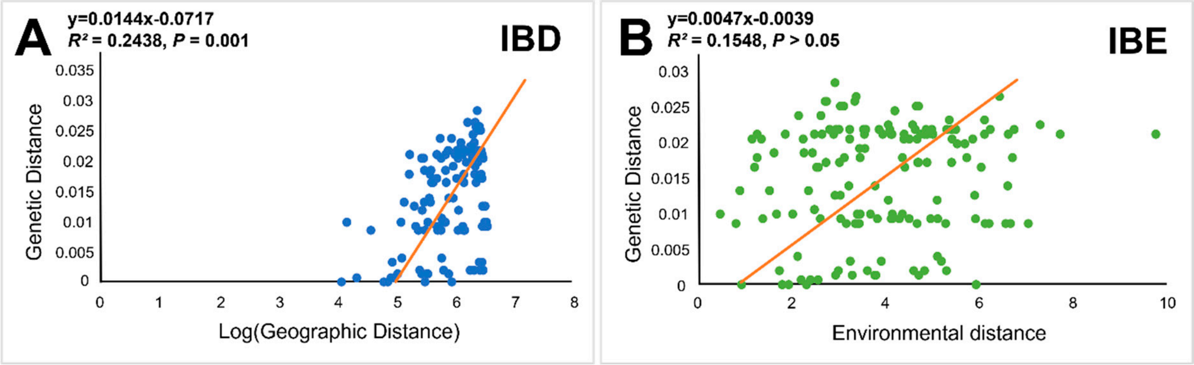

Figure S1. Results of the Mantel test of IBD (A) and IBE (B) analysis for all samples with precise location coordinates. Red lines show the trend of scatter plot.

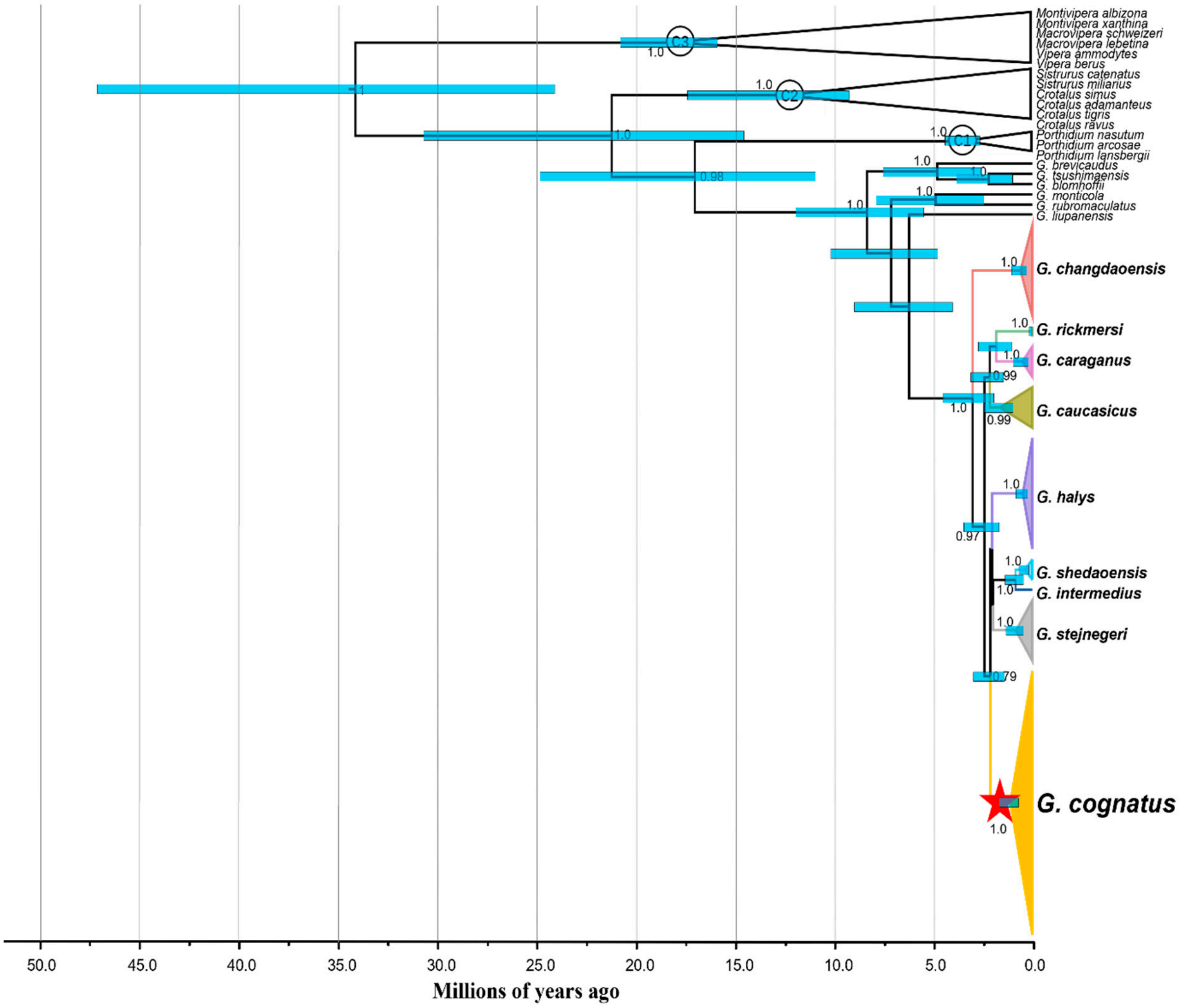

Figure S2. Molecular dating of the *G. halys*-*G. intermedius* complex based on the concatenated *ND4* and *Cytb* genes. Branch support values are not given for most intra-generic nodes to preserve clarity. C1, C2 and C3 indicate the calibration points placed on nodes, and red star denotes the node of *G. cognatus*, and bars show 95% HPD of divergence date.

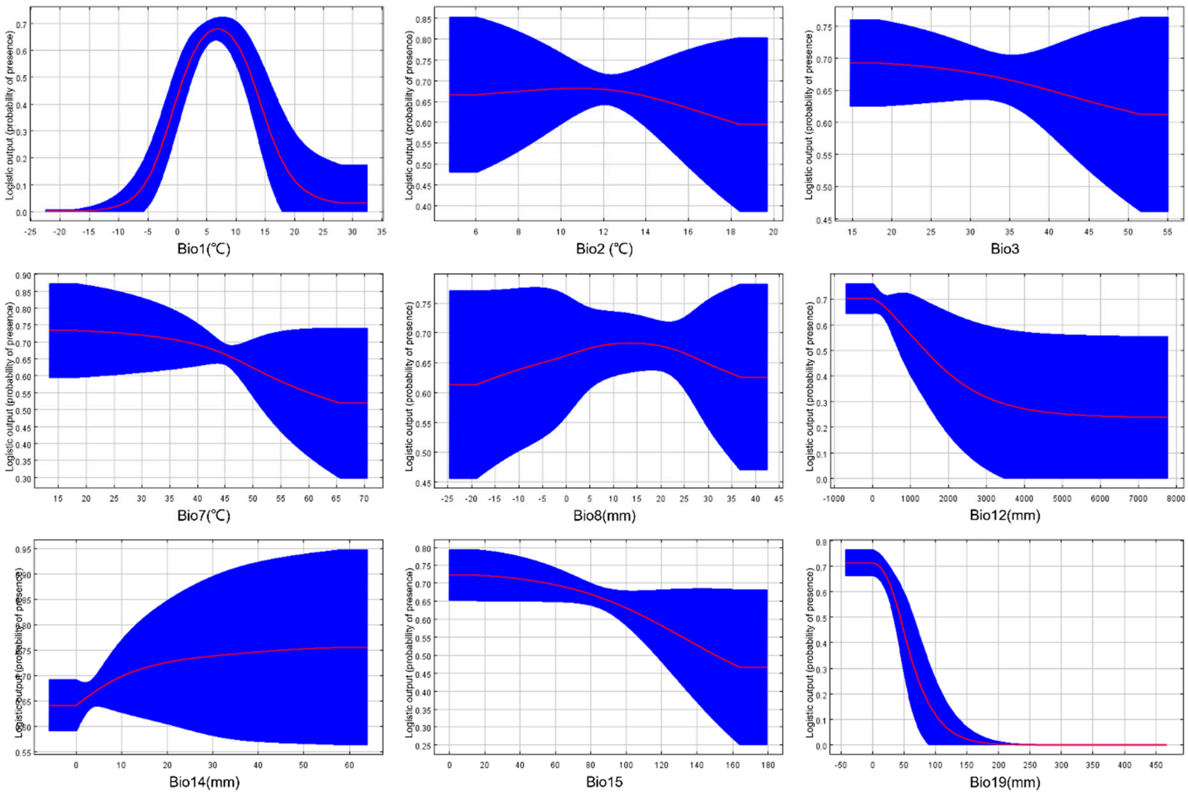

Figure S3. Average response curve profiles for the used variables in ENM.

Table S1. Samples information of the mtDNA data retrieved from GenBank.

Species	Site Number	Voucher Number	Code	Geographic Origin	Locality	Accession Number ND4	Accession Number Cytb	Reference
G. cognatus	13	GS070801	GS070801	Qaidam Basin	Delingha, Qinghai, China	KX063784	KX063811	[42]
	14	CIBQY224	QY224	Zoige Basin	Zoige, Sichuan, China	KY040640	KY040619	[43]
	14	CIBQY225	QY225	Zoige Basin	Zoige, Sichuan, China	KY040641	KY040620	[43]
	15	JS130947	47	Loess Plateau	Yinchuan, Ningxia, China	KY040643	KY040622	[43]
	16	JSSD1505N2	JSN2	Loess Plateau	Wuzhong, Ningxia, China	KX063781	KX063808	[42]
	16	JSSD1505N6	JSN6	Loess Plateau	Wuzhong, Ningxia, China	KX063782	KX063809	[42]
	17	JS15100005	05	Lanzhou Basin	Gansu, China	KX063787	KX063814	[42]
	18	BYEB1501	IA	Mongolian Plateau	Baotou, Inner Mongolia, China	KX063785	KX063812	[42]
	19	JSSD13109I1	I1	Mongolian Plateau	Sonid Right Banner, Inner Mongolia, China	KX063783	KX063810	[42]
	20	JSSD13109I3	I3	Mongolian Plateau	Inner Mongolia, China	KY040642	KY040621	[43]
	21	CHS098	CHS098	Mongolian Plateau	Inner Mongolia, China	–	MK201265	[56]
	22	GP188	GP188	Ily Basin	Xinjiang, China	JQ687469	JQ687488	[55]
	23	DLG21	G21	Ily Basin	Xinjiang, China	KX063786	KX063813	[42]
	24	CHS825	CHS825	Junggar Basin	Bole, Xinjiang, China	–	MK201554	[56]
G. halys		GP465	GP465		Xinjiang, China	JQ687475	JQ687494	[46
		JS1607H9	H9		Heilongjiang, China	KY040639	KY040618	[42]
		SYNU1510151	H2		Tahe, Heilongjiang, China	KX063773	KX063800	[42]
		SYNU1510154	H8		Huma, Heilongjiang, China	KX063774	KX063801	[42]
		SYNU1301908	46		Lingyuan, Liaoning, China	KX063775	KX063802	[42]
		JSSD1508X3	X3		Xilinhot, Inner Mongolia, China	KX063776	KX063803	[42]
		JSSD140M1	M1		Sunwu, Heilongjiang, China	KX063777	KX063804	[42]
		DLG11	G11		Xinglong, Hebei, China	KX063778	KX063805	[42]
		DLG12	G12		Chifeng, Inner Mongolia, China	KX063779	KX063806	[42]
		DLHSKG50	G50		Kazakhstan	KX063780	KX063807	[42]
G. stejnegeri		SYNU1510145	53		Mentougou, Beijing, China	KX063788	KX063815	[42]
		SYNU1510134	S1		Baoji, Shaanxi, China	KX063789	KX063816	[42]
		JSSD1409S3	S3		Tongchuan, Shaanxi, China	KX063790	KX063817	[42]
		JSSD1508S4	S4		Linfen, Shanxi, China	KX063791	KX063818	[42]
		JS151054	54		Beijing, China	KY040646	KY040625	[43]
		CHS323	CHS323		Nanniwan, Shaanxi, China	–	MK201393	[56]
		CHS322	CHS322		Yan'an, Shaanxi, China	–	MK201392	[56]
G. intermedius			KM434236		Heilongjiang, China	KM434236	KM434236	Xu and Zhao (Unpubl.)
		NIBR0000625204	NIBR		Gangwondo, South Korea	MW143075	MW143075	[112]
G. shedaoensis		JSSD1110D2	D2		Lvshun, Liaoning, China	KX063792	KX063819	[42]
		1102	1102		Changdao, Shandong, China	KT726956	KT726956	[113]
		1119	1119		Weihai, Shandong, China	KT726957	KT726957	[113]
G. qianshanensis		JSSD1110Q8	Q8		Pulandian, Liaoning, China	KX063793	KX063820	[42]
G. changdaoensis		JSSD1408Z1	Z1		Lianyungang, Jiangsu, China	KX063794	KX063821	[42]
		JSSD1510Z2	Z2		Lianyungang, Jiangsu, China	KX063795	KX063822	[42]

	JSSD1510C1	C1	Changdao, Shandong, China	KX063796	KX063823	[42]
	SYNU1510149_27	27	Weihai, Shandong, China	KX063797	KX063824	[42]
G. lijianlii	HS11047	HS11047	Jiadao, Shandong, China	KF997956	KF997904	Huang (Unpubl.)
	HS11048	HS11048	Xiaoheishandao, Shandong, China	KF997959	KF997905	Huang (Unpubl.)
	HS11052	HS11052	Miaodao, Shandong, China	KF997960	KF997906	Huang (Unpubl.)
	CHS084	CHS084	Yantai, Shandong, China	–	MK201256	[56]
	CHS085	CHS085	Yantai, Shandong, China	–	MK201257	[56]
	CHS326	CHS326	Jia Island, Shandong, China	–	MK201394	[56]
G. caraganus	JS1610CR2	CR2		MF490454	MF490456	[43]
	JS1610CR1	CR1		MF490453	MF490455	[43]
	RIZ20426.1	426	Kyzylorda, Kazakhstan	MZ959158	MZ959165	[114]
G. caucasicus	JS-2313	JS2313	Tajikistan	KY040656	KY040637	Shi (Unpubl.)
	RIZ29913	913	Mazandaran, Iran	MZ959159	MZ959166	[114]
	NEZMUT_1297	NE97	Alborz, Iran	MH378764	MH378727	[33]
	NEZMUT_1299	NE99	Alborz, Iran	MH378765	MH378728	[33]
	NEZMUT_61	NE61	Alborz, Iran	MH378729	MH378692	[33]
G. rickmersi	MHNG 2752.69	R1	Kyrgyzstan	KM078592	-	[43]
	MHNG 2752.70	R2	Kyrgyzstan	KM096379	-	[43]
G. monticola	GY001	GY001	Lijiang, Yunnan, China	JX661243	JX661200	Wu et al. (Unpubl.)
G. liupanensis	DLG5	G5	Ningxia, China	KX063798	KX063825	[42]
G. brevicaudu	DW005	DW005	Yantai, Shandong, China	JX661226	JX661199	Wu et al. (Unpubl.)
G. rubromaculatus	Y2	Y2	Yushu, Qinghai, China	KY040654	KY040633	[42]
G. blomhoffi	B524	B524	Japan	AY352814	AY352751	[115]
G. tsushimaensis	Ts1	Ts1	Japan	JN870211	JN870203	[116]
Sistrurus catenatus				AY223648	AY223610	[117]
Sistrurus miliarius				U41889	AY223611	[117,118]
Crotalus adamanteus				U41880	AY223605	[117,118]
Crotalus ravus				AY223647	AY223609	[119]
Crotalus simus				AY704885	AY704835	[120]
Crotalus tigris				AF156574	AY223606	[119,121]
Porthidium arcosae				AF292613	AF292575	[122]
Porthidium lansbergii				AF393623	AY713375	[122]
Porthidium nasutum				U41887	AY223579	[117,118]
Vipera berus				–	DQ186079	[123]
Vipera ammodytes				EU624232	DQ186513	[120,2]
Macrovipera lebetina				–	KJ415301	[124]
Macrovipera schweizeri				–	AJ275715	[125]
Montivipera albizona				–	KX168728	[126]
Montivipera xanthina				–	KX168811	[126]

References

2. Ursenbacher, S.; Schweiger, S.; Tomović, L.; Crnobrnja-Isailović, J.; Fumagalli, L.; Mayer, W. Molecular phylogeography of the nose-horned viper (*Vipera ammodytes*, Linnaeus (1758)): evidence for high genetic diversity and multiple refugia in the Balkan peninsula. *Mol. Phylogenet. Evol.* **2008**, *46*, 1116–1128. doi:10.1016/j.ympev.2007.11.002.

33. Asadi, A.; Montgelard, C.; Nazarizadeh, M.; Moghaddasi, A.; Fatemizadeh, F.; Simonov, E.; Kami, H.G.; Kaboli, M. Evolutionary history and postglacial colonization of an Asian pit viper (*Gloydius halys caucasicus*) into Transcaucasia revealed by phylogenetic and phylogeographic analyses. *Sci. Rep.* **2019**, *9*, 1224. doi:10.378/s41598-018-37558-8.

37. Wagner, P.; Tiutenko, A.; Mazepa, G.; Borkin, L.J.; Simonov, E. Alai! Alai! - a new species of the *Gloydius halys* (Pallas, 1776) complex (Viperidae, Crotalinae), including a brief review of the complex. *Amphib. Reptil.* **2016**, *37*, 15–31. doi:10.1163/15685381-00003026.

42. Shi, J.S.; Yang, D.W.; Zhang, W.Y.; Qi, S.; Li, P.P.; Ding, L. Distribution and intraspecies taxonomy of *Gloydius halys-Gloydius intermedius* complex in China (Serpentes: Crotalinae). *Chin. J. Zool.* **2016**, *51*, 777–798. doi:10.13859/j.cjz.201605008 (In Chinese with English abstract).
43. Shi, J.; Wang, G.; Fang, Y.; Ding, L.; Huang, S.; Hou, M.; Liu, J.; Li, P. A new moth-preying alpine pit viper species from Qinghai-Tibetan Plateau (Viperidae, Crotalinae). *Amphib. Reptil.* **2017**, *38*, 517–532. doi:10.1163/15685381-00003134.
55. Xu, Y; Liu, Q; Myers, E.A.; Wang, L.; Huang, S.; He, Y.; Peng, P.; Guo, P. Molecular phylogeny of the genus *Gloydius* (Serpentes: Crotalinae). *Asian Herpetol. Res.* **2012**, *3*, 127–132. doi:10.3724/SP.J.1245.2012.00127.
56. Li, J.N.; Liang, D.; Wang, Y.Y.; Guo, P.; Huang, S.; Zhang, P. A large-scale systematic framework of Chinese snakes based on a unified multilocus marker system. *Mol. Phylogenet. Evol.* **2020**, *148*, 106807. doi:10.1016/j.ympev.2020.106807.
112. Lee, Y.S.; Do, M.S.; Jeon, H.S.; Lee, S.C.; An, J. Complete mitochondrial genome of *Gloydius saxatilis* (Viperidae: crotalinae) from Korea. *Mitochondrial DNA B* **2021**, *6*, 645–647. doi:10.1080/23802359.2021.1878957.
113. Liu, Q.; Zhu, F.; Wang, X.; Xiao, R.; Fang, M.; Sun, L.; Li, P.; Guo, P. The complete mitochondrial genome sequence of *Gloydius shedaoensis* (Squamata: Viperidae). *Mitochondrial DNA A* **2016**, *27*, 4679–4680. doi:10.3109/19401736.2015.1106499.
114. Shi, J.S., Liu, J.C.; Giri, R.; Owens, J.B.; Santra, V.; Kuttalam, S.; Selvan, M.; Guo, K.J.; Malhotra, A. Molecular phylogenetic analysis of the genus *Gloydius* (Squamata, Viperidae, Crotalinae), with description of two new alpine species from Qinghai-Tibet Plateau, China. *ZooKeys*, **2021**, *1061*, 87. doi:10.3897/zookeys.1061.70420.
115. Malhotra, A.; Thorpe, R.S. A phylogeny of four mitochondrial gene regions suggests a revised taxonomy for Asian pitvipers (*Trimeresurus* and *Ovophis*). *Mol. Phylogenet. Evol.* 2004, 32, 83–100. doi:10.1016/j.ympev.2004.02.008.
116. Fenwick, A.M.; Greene, H. W.; Parkinson, C.L. The serpent and the egg: unidirectional evolution of reproductive mode in vipers? *J. Zoolog. Syst. Evol. Res.* **2012**, *50*, 59–66. doi:10.1111/j.1439-0469.2011.00646.x.
117. Parkinson, C.L.; Campbell, J.A.; Chippindale, P.T.; Schuett, G. Multigene phylogenetic analysis of pitvipers, with comments on their biogeography. In: Schuett, G.W.; Höggren, M.; M.E.; Greene, H.W. (Eds.). *Biology of the Vipers*; Eagle Mountain Publishing, Eagle Mountain, UT, 2002; pp. 93–110.
118. Kraus, F.; Mink, D.G.; Brown, W.M. Crotaline intergeneric relationships based on mitochondrial DNA sequence data. *Copeia* **1996**, *1996*, 763–773. doi: 10.2307/1447637.
119. Wüster, W.; Ferguson, J.E.; Quijada-Mascareñas, J.A.; Pook, C.E.; Salomão M.D.G.; Thorpe, R. S. Tracing an invasion: landbridges, refugia, and the phylogeography of the Neotropical rattlesnake (Serpentes: Viperidae: *Crotalus durissus*). *Mol. Ecol.* **2005**, *14*, 1095–1108. doi:10.1111/j.1365-294X.2005.02471.x.
120. Wüster, W.; Peppin, L.; Pook, C.E.; Walker, D.E. A nesting of vipers: phylogeny and historical biogeography of the Viperidae (Squamata: Serpentes). *Mol. Phylogenet. Evol.* **2008**, *49*, 445–459. doi:10.1016/j.ympev.2008.08.019.
121. Parkinson, C.L.; Zamudio, K.R.; Greene, H.W. Phylogeography of the pitviper clade *Agkistrodon*: historical ecology, species status, and conservation of cantils. *Mol. Ecol.* **2000**, *9*, 411–420. doi:10.1046/j.1365-294x.2000.00854.x.
122. Wüster, W.; Salomão, M.D.G.; Quijada-Mascareñas, J.A.; Thorpe, R.S.; Duckett, G.J.; Puorto, M.G.; Theakston, R.D.G.; Thorpe, R.S.; Warrell, D.A. Origins and evolution of the South American pitviper fauna: Evidence from mitochondrial DNA sequence analysis. In: Schuett, G.W.; Höggren, M.; M.E.; Greene, H.W. (Eds.). *Biology of the Vipers*; Eagle Mountain Publishing, Eagle Mountain, UT, 2002; pp. 111–128.
123. Ursenbacher, S.; Carlsson, M.; Helfer, V.; Tegelström, H.; Fumagalli, L. Phylogeography and Pleistocene refugia of the adder (*Vipera berus*) as inferred from mitochondrial DNA sequence data. *Mol. Ecol.* **2006**, *15*, 3425–3437. doi:10.1111/j.1365-294X.2006.03031.x.
124. Zinenko, O.; Stümpel, N.; Mazanaeva, L.; Bakiev, A.; Shiryaev, K.; Pavlov, A.; Kotenko, T.; Kukushkin, O.; Chikin, Y.; Duisebayeva,T.; ; Nilson, G.; Orlov, N.L.; Tuniyev, S.; Ananjeva, N.B.; Murphy, R.W.; Joger, U. Mitochondrial phylogeny shows multiple independent ecological transitions and northern dispersion despite of Pleistocene glaciations in meadow and steppe vipers (*Vipera ursinii* and *Vipera renardi*). *Mol. Phylogenet. Evol.* **2015**, *84*, 85–100. doi:10.1016/j.ympev.2014.12.005.
125. Lenk, P.; Kalyabina, S.; Wink, M.; Joger, U. Evolutionary relationships among the true vipers (Reptilia: Viperidae) inferred from mitochondrial DNA sequences. *Mol. Phylogenet. Evol.* **2001**, *19*, 94–104. doi:10.1006/mpev.2001.0912.
126. Stümpel, N.; Rajabizadeh, M.; Avcı, A.; Wüster, W.; Joger, U. Phylogeny and diversification of mountain vipers (*Montivipera*, Nilson et al., 2001) triggered by multiple Plio-Pleistocene refugia and high-mountain topography in the Near and Middle East. *Mol. Phylogenet. Evol.* **2016**, *101*, 336–351. doi:10.1016/j.ympev.2016.04.025.

Table S2. Data on the phylogenetic, molecular dating and BSP analyses, including partitions, models and parameters.

Analysis	Software	Dataset	Best model	Partition identity	Length (bp)	Clock model	Tree model (linked)	Run specifications
Phylogenetic analysis: Bayesian Inference	MrBayes	All sequences in *G. cognatus* and two outgroups	TRN+I+G	*ND4*-1st, *ND4*-2nd, *Cytb*-1st, *Cytb*-2nd	1194			2 runs; 2×10^7 generations; 1000 sampling frequency; 25% burn-in
			GTR+G	*ND4*-3rd, *Cytb*-3rd	597			
Phylogenetic analysis: Maximum Likelihood	IQ-TREE	All sequences in *G. cognatus* and two outgroups	TRN+I+G	*ND4*-1st, *ND4*-2nd, *Cytb*-1st, *Cytb*-2nd	1194			Ultrafast-bootstrap (UFBoot) ×10,000
			GTR+G	*ND4*-3rd, *Cytb*-3rd	597			
Molecular dating with calibration points	BEAST	All sequences of *G. halys-G. intermedius* complex and 21 outgroups	TRN+I+G	*ND4*-1st, *ND4*-2nd, *Cytb*-1st, *Cytb*-2nd	1194	Relaxed Uncorrelated Lognormal	Birth-and-death Random starting tree	2 runs; 4×10^7 generations; 1000 sampling frequency; 25% burn-in
			GTR+G	*ND4*-3rd, *Cytb*-3rd	597			
Molecular dating with constant-size tree prior	BEAST	All sequences in *G. cognatus*	TRN+I+G	*ND4*-1st, *ND4*-2nd, *Cytb*-1st, *Cytb*-2nd	1194	Relaxed Uncorrelated Lognormal	Constant-size Coalescence Random starting tree	2 runs; 1×10^7 generations; 1000 sampling frequency; 25% burn-in
			GTR+G	*ND4*-3rd, *Cytb*-3rd	597			

BSP analysis	BEAST	All sequences in South Clade	TRN+I+G	*ND4*-1st, *ND4*-2nd, *Cytb*-1st, *Cytb*-2nd	1194	Strict clock	Coalescent: Bayesian Skyline Plot Random starting tree	2 runs; 4×10^7 generations; 5000 sampling frequency; 25% burn-in
			GTR+G	*ND4*-3rd, *Cytb*-3rd	597			
BSP analysis	BEAST	All sequences in North Clade	TRN+I+G	*ND4*-1st, *ND4*-2nd, *Cytb*-1st, *Cytb*-2nd	1194	Strict clock	Coalescent: Bayesian Skyline Plot Random starting tree	2 runs; 4×10^7 generations; 5000 sampling frequency; 25% burn-in
			GTR+G	*ND4*-3rd, *Cytb*-3rd	597			

Table S3. The 18 sampling sites used for IBD and IBE analysis.

Site Number	Voucher Number	Location	Latitude (N)	Longitude (E)
1	KZ26	Raiymbek District, Almaty Region, Kazakhstan	43.16	79.21
2	KZ08	Raiymbek District, Almaty Region, Kazakhstan	42.97	79.31
3	CKG041	Raiymbek District, Almaty Region, Kazakhstan	43.02	79.89
4	CKG036	Uygur District, Almaty Region, Kazakhstan	43.43	80.07
5	XU202105	Gongliu County, Xinjiang, China	43.26	82.09
6	Guo4708	Nilka County, Xinjiang	43.8	82.43
7	Guo2481	Wusu City, Xinjiang, China	45.05	84.66
8	GXG310	Hoboksar, Xinjiang, China	46.58	85.65
9	GXG807	Tsalgar, Uvs, Mongolia	49.37	92.93
10	Guo5242	Yongdeng County, Gansu, China	36.49	103.4
11	Guo2707	Jingyuan County, Gansu, China	36.55	104.68
12	Guo875	Otog Banner, Inner Mongolia, China	38.79	107.34
13	GS070801	Delingha City, Qinghai, China	37.38	97.40
13	GS070802	Delingha City, Qinghai, China	37.38	97.40
14	CIBQY224	Zoige, Sichuan, China	33.60	102.93
14	CIBQY225	Zoige, Sichuan, China	33.60	102.93
15	JS130947	Yinchuan, Ningxia, China	38.48	106.21
16	JSSD1505N2	Wuzhong, Ningxia, China	38.00	106.19
16	JSSD1505N6	Wuzhong, Ningxia, China	38.00	106.19
18	BYEB1501	Baotou, Inner Mongolia, China	40.48	109.84
19	JSSD13109I1	Sonid Right Banner, Inner Mongolia, China	42.74	112.62

Table S4. Locations and coordinates used for ENM.

Location	Latitude	Longitude	Reference	Year
Raiymbek District, Almaty Region, Kazakhstan	43.16	79.21	This study	2013
Raiymbek District, Almaty Region, Kazakhstan	42.97	79.31	This study	2013
Raiymbek District, Almaty Region, Kazakhstan	43.02	79.89	This study	2017
Uygur District, Almaty Region, Kazakhstan	43.43	80.07	This study	2017
Gongliu County, Xinjiang, China	43.26	82.09	This study	2021
Nilka County, Xinjiang	43.80	82.43	This study	2015
Wusu City, Xinjiang, China	45.05	84.66	This study	2013
Hoboksar, Xinjiang, China	46.58	85.65	This study	2019
Tsalgar, Uvs, Mongolia	49.37	92.93	This study	2019
Yongdeng County, Gansu, China	36.49	103.40	This study	2016
Jingyuan County, Gansu, China	36.55	104.68	This study	2013
Otog Banner, Inner Mongolia, China	38.79	107.34	This study	2010
Delingha City, Qinghai, China	37.38	97.40	This study	2007
Zoige, Sichuan, China	33.60	102.93	[43]	2017
Yinchuan, Ningxia, China	38.48	106.21	[43]	2017
Wuzhong, Ningxia, China	38.00	106.19	[42]	2016
Baotou, Inner Mongolia, China	40.48	109.84	[42]	2016
Sonid Right Banner, Inner Mongolia, China	42.74	112.62	[42]	2016
Qinghe County, Xinjiang, China	46.41	90.61	This study	2022/2023

References

42. Shi, J.S.; Yang, D.W.; Zhang, W.Y.; Qi, S.; Li, P.P.; Ding, L. Distribution and intraspecies taxonomy of *Gloydius halys-Gloydius intermedius* complex in China (Serpentes: Crotalinae). *Chin. J. Zool.* **2016**, *51*, 777–798. doi:10.13859/j.cjz.201605008 (In Chinese with English abstract).
43. Shi, J.; Wang, G.; Fang, Y.; Ding, L.; Huang, S.; Hou, M.; Liu, J.; Li, P. A new moth-preying alpine pit viper species from Qinghai-Tibetan Plateau (Viperidae, Crotalinae). *Amphib. Reptil.* **2017**, *38*, 517–532. doi:10.1163/15685381-00003134.

Table S5. Uncorrelated climatic factors used in ENM and their contribution rates.

ID	Environment Variable	Contribution Rates	Permutation Importance
Bio1	Annul mean temperature	55.8±18.05	43.8±13.45
Bio2	Mean diurnal range	2.5±2.61	2.1±3.17
Bio3	Isothermality	1.6±4.46	0.4±1.27
Bio7	Temperature annual range	2.0±3.30	3.7±5.82
Bio8	Mean temperature of wettest quarter	2.6±4.99	0.7±1.68
Bio12	Annual precipitation	1.2±2.80	3.7±5.43
Bio14	Precipitation of driest month	0.4±0.74	2.6±4.14
Bio15	Precipitation seasonality (coefficient of variation)	4.7±5.80	4.5±5.35
Bio19	Precipitation of coldest quarter	29.2±12.83	38.5±10.52

Table S6. Hierarchical analysis of AMOVA for *G. cognatus*.

**Hypothesized Structure**	**Among Groups**			**Among Populations Within Groups**			**Within Populations**		
	df	**%var**	***Fct* (*P*-value)**	***df***	**%var**	***Fsc* (*P*-value)**	***df***	**%var**	***Fst* (*P*-value)**
(N1, N2) vs. (S1, S2)	1	55.92	0.55919**	16	41.98	0.95242	3	2.1	0.97903**
N1 vs. N2 vs. S1 vs. S2	3	74.57	0.74565**	14	23.03	0.97598*	3	2.40	0.97598**

Significance level: 0.01*; 0.001**
